# Integrating DNA Barcoding and Traditional Taxonomy for the Identification of Dipterocarps in Remnant Lowland Forests of Sumatra

**DOI:** 10.3390/plants8110461

**Published:** 2019-10-30

**Authors:** Carina Carneiro de Melo Moura, Fabian Brambach, Kevin Jair Hernandez Bado, Konstantin V. Krutovsky, Holger Kreft, Sri Sudarmiyati Tjitrosoedirdjo, Iskandar Z. Siregar, Oliver Gailing

**Affiliations:** 1 Department of Forest Genetics and Forest Tree Breeding, University of Göttingen, Büsgenweg 2, 37077 Göttingen, Germany; carinamoura@uni-goettingen.de (C.C.d.M.M.); konstantin.krutovsky@forst.uni-goettingen.de (K.V.K.); 2 Biodiversity, Macroecology and Biogeography, University of Göttingen, Büsgenweg 1, 37077 Göttingen, Germany; fbrambach@uni-goettingen.de (F.B.); holger.kreft@forst.uni-goettingen.de (H.K.); 3 Center for Integrated Breeding Research, University of Göttingen, Albrecht-Thaer-Weg 3, 37075 Göttingen, Germany; 4 Laboratory of Population Genetics, N. I. Vavilov Institute of General Genetics, Russian Academy of Sciences, 3 Gubkin Str., Moscow 119333, Russian; 5 Laboratory of Forest Genomics, Genome Research and Education Center, Institute of Fundamental Biology and Biotechnology, Siberian Federal University, 50a/2 Akademgorodok, Krasnoyarsk 660036, Russia; 6 Department of Ecosystem Science and Management, Texas A&M University, College Station, TX 77843-2138, USA; 7 Southeast Asian Regional Center for Tropical Biology (SEAMEO BIOTROP), Jalan Raya Tajur Km. 6, Bogor 16144, Indonesia; sritjitro@gmail.com; 8 Department of Silviculture, Faculty of Forestry, Bogor Agricultural University, Dramaga Campus, Bogor 16680, Indonesia; izsiregar@gmail.com

**Keywords:** *matK*, *rbcL*, *trnL-F*, Dipterocarpoideae, tropical tree diversity, genetic distance, reference DNA library

## Abstract

DNA barcoding has been used as a universal tool for phylogenetic inferences and diversity assessments, especially in poorly studied species and regions. The aim of this study was to contrast morphological taxonomy and DNA barcoding, using the three frequently used markers *matK*, *rbcL*, and *trnL-F*, to assess the efficiency of DNA barcoding in the identification of dipterocarps in Sumatra, Indonesia. The chloroplast gene *matK* was the most polymorphic among these three markers with an average interspecific genetic distance of 0.020. The results of the molecular data were mostly in agreement with the morphological identification for the clades of *Anthoshorea*, *Hopea*, *Richetia*, *Parashorea*, and *Anisoptera*, nonetheless these markers were inefficient to resolve the relationships within the *Rubroshorea* group. The maximum likelihood and Bayesian inference phylogenies identified *Shorea* as a paraphyletic genus, *Anthoshorea* appeared as sister to *Hopea*, and *Richetia* was sister to *Parashorea*. A better discriminatory power among dipterocarp species provided by *matK* and observed in our study suggests that this marker has a higher evolutionary rate than the other two markers tested. However, a combination of several different barcoding markers is essential for reliable identification of the species at a lower taxonomic level.

## 1. Introduction

The Dipterocarpaceae family consists of approximately 680 species that are commonly placed in the two subfamilies, Monotoideae (~30 species in Africa and Madagascar, and the monotypic *Pseudomonotes* in Colombia) and Dipterocarpoideae, which hold the majority of species and are confined to the eastern tropics from India to New Guinea [1,2,3,4,5,6]. Members of the Dipterocarpoideae dominate the diverse rainforests of Sundaland, often with many co-occurring species [7]. Due to their high abundance, mechanical wood-properties, and tall stature, they provide some of the most valued and abundant sources of tropical hardwood, and at the same time are significant stores of aboveground carbon [8]. Hence, dipterocarps are of great ecological and economic importance [6], and understanding their diversity is necessary to advance our general knowledge of Southeast Asian rainforests. Despite being some of the most prevalent trees in the Asian tropics, dipterocarps are increasingly threatened by deforestation and land-use change [9]. For effective conservation of the threatened species and genetic diversity of the Dipterocarpaceae, it is important to understand their species-level taxonomy as well as their origin and the evolutionary processes that have led to the astonishing diversity of the family. To this end, molecular phylogenies are needed that provide basic knowledge on the evolutionary history and phylogenetic relationships of extant species [10]. Indeed, the dipterocarp family has already received great interest in traditional morphology-based taxonomy [11,12,13] and different phylogenetic studies based on DNA-markers ranging from Restriction Fragment Length Polymorphism (RFLP) of plastid regions [1], plastid DNA sequences [14,15], internal transcribed spacers [16], and nuclear genes [17] to genome-wide markers [3,18,19].

The exact taxonomic placement of the Dipterocarpaceae family within the angiosperms was disputed for a long time, including placements into the orders Theales and Malvales [20]. Based on recent phylogenetic and phylogenomic studies, the placement in Malvales is now universally accepted, but the relationships among the Dipterocarpaceae and the closely related families Cistaceae and Sarcolaenaceae are still insufficiently resolved [21]. The largest tribe of Dipterocarpaceae, Shoreeae, consists of the genera *Shorea*, *Hopea*, *Parashorea*, and the monotypic *Neobalanocarpus* [22]. Generic limits in the tribe are obscure and recent studies have shown that *Shorea* is in fact paraphyletic with *Hopea*, *Parashorea*, and *Neobalanocarpus* nested within it [3,23]. Nevertheless, based on phylogenomic data, well-defined clades within the tribe Shoreeae can be identified. They are consistent with most of the traditional genera and recognized subgenera of *Shorea*: *Anthoshorea* (white meranti), *Richetia* (yellow meranti), *Shorea* (balau/selangan batu), *Rubroshorea* (red meranti), and *Doona* (*Pentacme* has not been included in phylogenomic studies so far) [3,19].

Despite the comparatively large attention that this group has received due to its economic importance, identification of dipterocarps can be challenging [11,12,13]. This is due mainly to the large size of most dipterocarps and their characteristic periodic mass flowering and fruiting, which results in trees with reproductive structures absent for most of the time. Another complicating factor—especially in ecological studies—is that several closely related and morphologically similar species may co-occur. In this context, DNA barcoding can provide an independent source of information to delimit and identify species [24,25].

The *matK* and *rbcL* loci are considered standard plant DNA barcoding markers due to their universality, relatively high overall sequence quality, low cost, and high discriminatory power between angiosperms [26,27]. Specifically, *rbcL* has a higher PCR amplification success, but lower discriminatory power than *matK*. The use of both barcoding markers, *rbcL* together with *matK*, was proposed by the CBOL Plant Working Group of the Consortium for the Barcoding of Life [28] to have a higher combined discriminatory power [29]. Non-coding regions have also been implemented as barcoding marker [30]. Thus, joint use of coding and non-coding regions is an important step to implement a plant barcode database as a tool for accurate diversity assessments and to develop conservation strategies.

A detailed DNA barcoding dataset of dipterocarps with comprehensive coverage across taxonomic groups and geographic areas is currently lacking [25]. So far, most efforts have concentrated on the Malay Peninsula and Borneo [3,16,24], while material from Sumatra has hardly been included in analyses (but see [25]). At the same time, large-scale logging and subsequent deforestation in Sumatra over the last decades [31] have decimated dipterocarp populations on the island, and many of the formerly widespread species are now threatened with extinction [32]. Detailed assessments of dipterocarp diversity and composition in the remaining forests of Sumatra are therefore a requirement for effective conservation measures. Here, we contrast the traditional morphological taxonomy and the DNA barcoding approach for the identification of dipterocarp species in remnant lowland rainforests of Sumatra, Indonesia. We used three DNA barcoding markers—*rbcL*, *matK*, and *trnL-F* to (i) assess the dipterocarp identification using phylogenetic trees; and (ii) test the efficiency of these markers for the identification of dipterocarps.

## 2. Materials and Methods

### 2.1. Study Area and Specimen Collection

The study was a part of the Collaborative Research Centre 990: Ecological and Socio-economic Functions of Tropical Lowland Rainforest Transformation Systems (CRC990: EFForTS project, https://www.uni-goettingen.de/efforts) in Jambi Province, central Sumatra, Indonesia. The study region is characterized by an average annual temperature of 26.7 ± 0.2 °C and mean annual precipitation of approximately 2235 ± 381 mm [25,33,34]. Samples were collected in two areas, the ‘Bukit Duabelas landscape’ and the ‘Harapan landscape’, respectively, as part of plot based (0.25 ha, 50 × 50 m, four plots per landscape and land-use) inventories on well-drained soils in four land-use types: (1) logged-over primary rain forest, (2) jungle rubber agroforestry, (3) rubber plantations, and (4) oil palm plantations. In the ‘Harapan landscape’, we also collected samples from 12 riparian plots, four each in logged-over forest, rubber and oil palm plantations. Details about the sampling design can be found in Drescher et al. [33] and Paoletti et al. [35]. Over well-drained soils, dipterocarp species were abundant and diverse in all forest plots (c. 280 individuals in 13 species) but were mainly absent from the more intensely used land-use types (one species rarely present in rubber agroforestry, another in rubber monoculture) [34]. We collected a total of 80 herbarium specimens assigned initially to Dipterocarpaceae in the field.

### 2.2. Morphology-Based Species Identification

**Figure 1:**
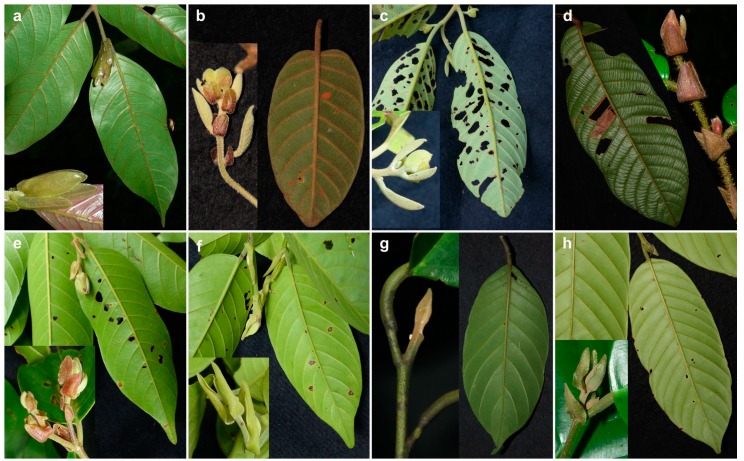
Morphological traits of all Sumatran species and subspecies of red meranti (*Shorea* sect. *Rubroshorea*) sampled for this study. Shown are branchlets, stipules, and leaves of (**a**) *Shorea acuminata* (from specimen Rembold KR0822), (**b**) *S. dasyphylla* (KR0546), (**c**) *S. leprosula* (KR5454), (**d**) *S. ovalis* (KR0891), (**e**) *S. parvifolia* subsp. *parvifolia* (KR5463), (**f**) *S. parvifolia* subsp. *velutinata* (KR5509), (**g**) *S. pauciflora* (KR4807), and (**h**) *S. singkawang* (KR0842). Not to scale, photographs by K. Rembold.

During plot-inventories, all species were pre-identified as morphospecies in the field. For each morphospecies, herbarium specimens of at least one individual were collected, stored and prepared for later morphological identification at Indonesian herbaria (Herbarium Bogoriense and BIOTROP Herbarium). The herbarium specimens were cross-referenced with the available specimens at the Indonesian herbaria and identified to species or morphospecies level by associated taxonomists. Subsequently, we checked and revised all identifications by comparing collected specimens and high-quality standardized photographs taken in the field to keys and descriptions in standard taxonomic literature [11,12,13,36] and the online repositories of herbarium specimens at BioPortal (http://bioportal.naturalis.nl) and JSTOR Global Plants (http://plants.jstor.org). During the identification, we focused on vegetative traits, as flowering or fruiting material was not available. Species were distinguished based on traits of the trunk (e.g., presence/form of buttresses and stilt-roots), bark (including the inner layers), twigs and stipules (size, color, indument), and leaves (petiole, size, venation, surface, indument). Representative specimens for all species of *Shorea* sect. *Rubroshorea* are shown in Figure 1. We also checked the identifications based on morphology against the placement of all specimens in our phylogenetic trees.

### 2.3. DNA Extraction, PCR Amplification, and Sequencing

Together with herbarium specimens, leaf tissues of approximately 2 cm^2^ were collected from each sample and dried in silica-gel until DNA extraction. DNA was extracted from the dried leaf tissue following the manufacturer’s protocol for the DNeasy 96 Plant Mini Kit (Qiagen, Hilden, Germany). The concentration of the extracted DNA was checked using 1% agarose gel electrophoresis with 1X TAE buffer solution, and 4 µL Roti-Safe dye. DNA fragments for each sample were then isolated and purified from the agarose gel with a volume of 13 µL Elution Buffer (innuPREP Gel Extraction Kit, Analytik Jena, Jena, Germany).

For each extracted DNA sample, polymerase chain reaction (PCR) was carried out using universal primers for the chloroplast DNA markers *rbcL*, *matK*, and *trnL-F* (Table 1). PCR was performed in a Peltier Thermal Cycler PTC-200 (MJ Research Inc., Waltham, MA, USA) with a total reaction mixture volume of 14 μL, which included a diluted 1 μL DNA sample, 1.5 µL PCR buffer (with 0.8 M Tris-HCl, 0.2 M (NH_4_)2SO_4_), 1.5 µL MgCl_2_ (25 mM), 1 µL dNTPs (2.5 mM of each dNTP), 1 µL of forward primer, and 1 µL reverse primer (5 pM/µL each), 0.2 µL (5 U/µL) HOT FIREPol^®^ Taq-Polymerase (Solis BioDyne, Tartu, Estonia), and 6.8 µL ddH_2_O.

The PCR program consisted of an initial denaturation at 95 °C for 15 min, followed by 35 cycles of denaturation at 94 °C for 1 min, annealing at 50 °C for 1 min, elongation at 72 °C for 1.5 min and a final extension at 72 °C for 20 min. PCR products were separated and visualized on 1% agarose gels, excised from the gel and purified with the innuPREP Gel Extraction Kit protocol (Analytik Jena, Jena, Germany).

Sequencing reactions were done with the BrilliantDye v3.1 Terminator Cycle Sequencing Kit optimized for Dye Set Z (NIMAGEN, Nijmegen, The Netherlands), and purified following the manufacturer’s protocol of DyeEx^®^ 96 Kit (Qiagen, Hilden, Germany). The same primers used for amplification were also used for sequencing (Table 1). The total sequencing reaction mixture included 2 μL DNA template (5–10 ng), 4.5 μL ddH_2_O, 0.5 μL BrilliantDye v3.1, 2 μL 5X Sequencing Buffer, 1 μL Forward/Reverse primer (5 pM/µL). Nucleotide sequences were analyzed using an ABI Prism Genetic Analyzer 3130xl with the Sequence Analysis v5.3.1 software (Applied Biosystems, Foster City, CA, USA).

### 2.4. Nucleotide Sequence Data Analysis

Both forward and reverse nucleotide sequence were visualized and aligned using the CodonCode Aligner software (https://www.codoncode.com/aligner). Sequences were manually checked; sequencing errors, if any, were corrected, consensus sequences were generated and then used for multiple sequence alignments. BLAST searches were performed for consensus sequences to identify best matches in the National Center for Biotechnology Information (NCBI) GenBank and Barcode of Life Data Systems (BOLD) [41] databases. Additionally, sequences from BOLD were included in the phylogenetic reconstruction (accession numbers are presented in the figures). All amplified sequences of the Dipterocarpaceae family obtained in this study were uploaded to the NCBI Genbank database, accession numbers MN444889-MN445045.

### 2.5. Genetic Distance and Phylogenetic Analysis

The nucleotide divergence between sequences was estimated using the Kimura-2-parameter genetic distance for each barcode markers *matK*, *rbcL* and *trnL-F* and for the combined markers (*matK* + *rbcL* and *matK* + *rbcL + trnL-F*) using their concatenated sequences. A uniform distribution was set as rate variation among sites. The overall mean genetic distance, as well as intraspecific and interspecific genetic distances were calculated for each species identified by traditional taxonomical features.

Phylogenetic trees were generated for each marker separately and based on the three markers combined (total length of alignment = 2204 bp; *matK* = 614 bp; *rbcL* = 603; *trnL-F* = 987 bp; see Table 2) using maximum likelihood (ML) methods in MEGA-X software [42] and Bayesian inference in BEAST and BEAUti 1.8.0 [43] by choosing the Hasegawa, Kishino and Yano (HKY) model as a nucleotide substitution model for nucleotide sites, “Yule process” option (Yule model of branching) for trees and “strict model” for molecular clock that assumes homogeneous rates among branches [44]. The HKY model considers different rates of transitions and transversions as well as unequal frequencies [45]. The considered rate of variation among sites for this model was the gamma distribution with five discrete gamma categories. Stationarity and convergence of runs were checked using Tracer 1.5 [46]. The maximum clade credibility tree was generated from trees produced by BEAST using TreeAnnotator 1.8.0 [43]. ML trees were calculated with 1000 bootstrap replications using the HKY model. The initial tree for the ML tree was kept as default preference, and the nearest-neighbor-interchange (NNI) heuristic method was used to search for the final ML tree. Gaps and missing data treatment were selected as partial deletion with 95% site coverage cutoff.

## 3. Results

### 3.1. Taxonomic Resolution of DNA Barcoding Markers

The sequencing success rate of the Dipterocarpaceae family for *matK*, *rbcL*, and *trnL-F* markers was 81%, 83.7%, and 54%, respectively (Table 2). The results of the BLAST performed using NCBI and BOLD platforms allowed us to correct the taxonomic identification for a significant number of specimens. Thirty-seven percent of the species, 5% of genera and 4% of all families were reassigned after comparison with the barcoding dataset and based on the subsequent new morphological identification performed using phylogenies as support (see Table S1).

The *matK* marker was efficient to identify samples at species level for the specimens belonging to the groups *Anthoshorea*, *Hopea*, *Richetia*, and *Parashorea*. However, this marker proved inefficient to resolve the relationships within the *Rubroshorea* clade.

**Figure 2:**
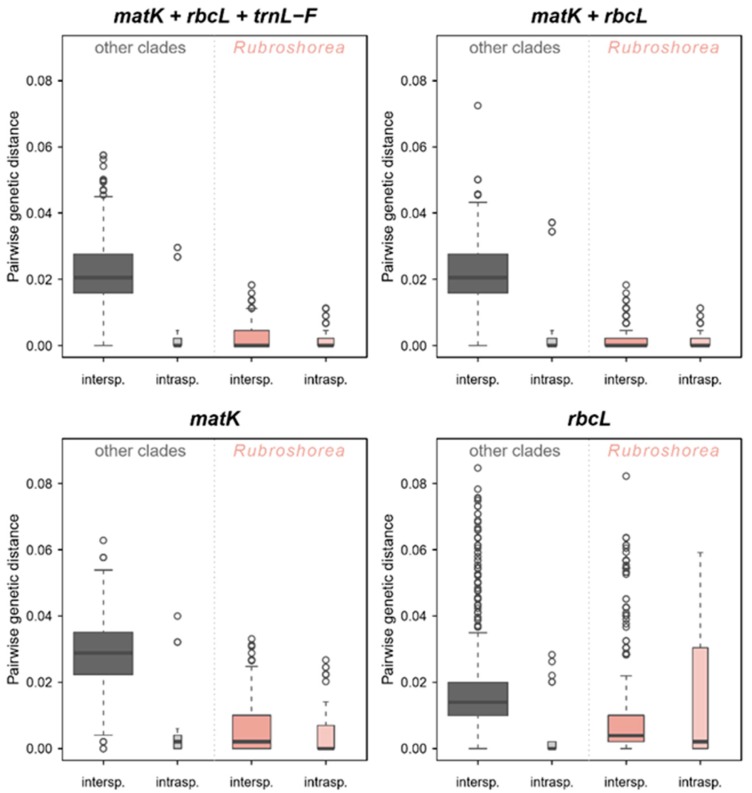
Boxplot representation of intraspecific (intrasp.) and interspecific (intersp.) pairwise genetic distances for the Dipterocarpaceae family based on each traditional barcode marker and the combined dataset: *matK* + *rbcL* + *trnL-F*; *rbcL* + *matK*; *matK;* and *rbcL*. *Rubroshorea* was plotted separately since the barcode makers used in this study failed to differentiate this section at species level. The width of each boxplots is proportional to the square-roots of the number of observations.

The overall genetic distance estimated for the *matK* sequences was 0.020, for *rbcL* 0.017, for *trnL-F* 0.026, for both *matK* and *rbcL* 0.019, and 0.021 for the three barcodes together. Figure 2 shows the boxplots of the genetic distances, revealing a clear difference between the intraspecific and interspecific genetic distances for each barcode marker and for the combined dataset (*matK* + *rbcL* + *trnL–F* and *matK* + *rbcL*) for all clades except *Rubroshorea*. Low differences were observed between pairwise intra- and interspecific genetic distances within section *Rubroshorea*, as the barcode makers used in this study were unsuccessful to distinguish the section *Rubroshorea* at species level (Figure 2).

### 3.2. Species Assignment Using Phylogenetic Trees

**Figure 3:**
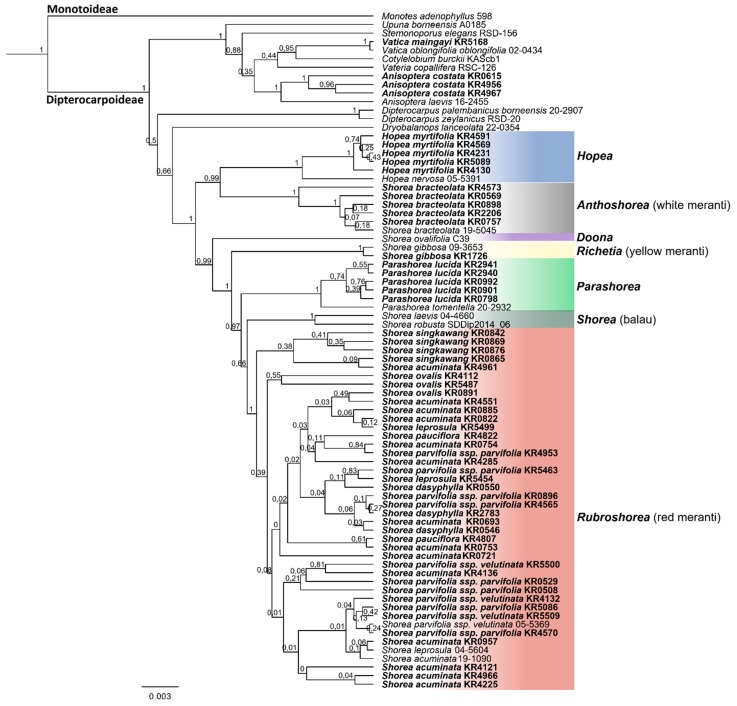
Bayesian inference tree based on the concatenated sequences of the *matK*, *rbcL*, and *trnL*-*F* markers. The numbers at the tree nodes represent the posterior probability. Tips display species IDs, samples collected for this study are depicted in bold face (see Table S1 for details), major clades of Shoreeae are color-highlighted.

**Figure 4:**
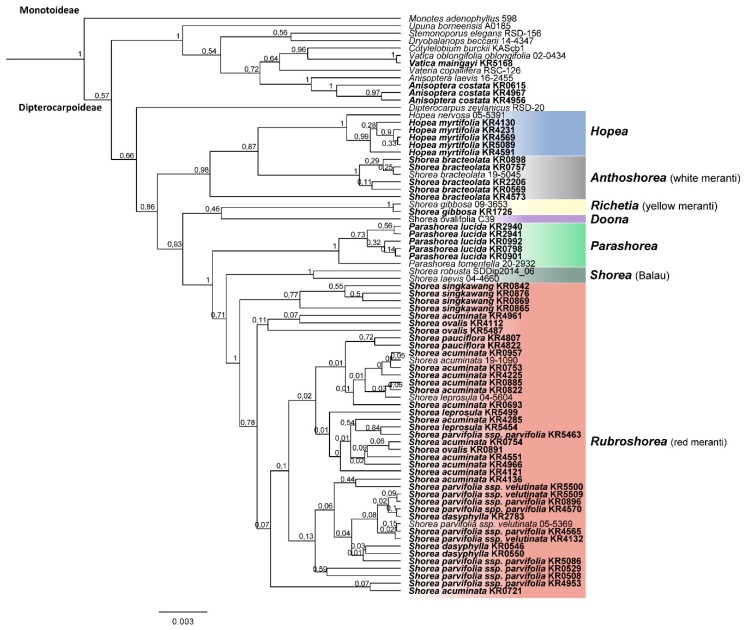
Bayesian inference tree based on the concatenated sequences of the *matK* and *rbcL* markers. The numbers at the tree nodes represent the posterior probability. Tips display species IDs, samples collected for this study are depicted in bold (see Table S1 for details), major clades of Shoreeae are color-highlighted.

All markers were efficient to distinguish the taxa at the family level, and the combination of the DNA barcodes was more efficient to allow the taxonomic identification of the Dipterocarpaceae at lower taxonomic levels (Table 3). In all phylogenetic trees (Figure 3 and Figure 4, and Figures S1–S4), the phylogenetic relationships within *Rubroshorea* remained unresolved.

The topology of the BI and ML trees mirrored each other, the main clades presented good support (bootstrap/posterior probabilities > 0.7/70%) and were consistent for each marker and for the concatenated sequences. *Monotes* was set as outgroup in the phylogenetic analysis (Figure 3 and Figure 4, and Figures S1–S4).

Overall, the phylogenetic tree based on the sequences of the three concatenated markers showed stronger node support and better resolution of the relationships between species of the Dipterocarpaceae family than the individual markers (Table 3). Dipterocarpoideae was resolved as monophyletic lineage with strong support (PP = 1). *Vatica* and *Anisoptera* were retrieved with strong support (PP = 0.95 and 1, respectively, Figure 3 and Figure S1) and *Dryobalanops* was sister to Shoreeae with moderate support (PP = 0.66). Paraphyletic *Shorea* (including *Hopea* and *Parashorea*) was divided into seven major lineages with high posterior probabilities (0.97 to 1.0, Figure 3 and Figure S1): *S. bracteolata* (*Shorea* subgenus *Anthoshorea*) and *Hopea* appeared as sister to the remaining clades. *Shorea* subgenus *Doona*, subgenus *Richetia*, *Parashorea*, and subgenus *Shorea* (Balau lineage) were then successively sister to the large subgenus *Rubroshorea*. The latter comprised a monophyletic group, but resolution within the clade was low (Figure 3 and Figure S1). The combined tree and the individual markers failed to resolve the taxonomic relationship within *Rubroshorea*, but were efficient to assess a precise taxonomic identification at species level for the following taxa: *Anisoptera costata*, *Vatica maingayi*, *Hopea myrtifolia*, *Shorea bracteolata*, *Shorea gibbosa,* and *Parashorea lucida*.

The resolution of the two-marker tree based on *rbcL* and *matK* was equivalent to the tree using the additional intergenic spacer *trnL-F*, and grouped all main lineages in monophyletic clades, however with lower bootstrap support for the Shoreeae clade (PP = 0.86). Nevertheless, low posterior probability was found supporting the lineages Balau, *Parashorea,* and *Richetia* (Figure 4). The combination of *matK* and *rbcL* was efficient to identify the following taxa at species level: *A. costata*, *V. oblongifolia, H. myrtifolia, S. bracteolata, P. lucida, S. gibbosa,* and *S. singkawang.*

Overall, the phylogenetic tree based on the *matK* sequences (Figure S2) displayed a similar topology in comparison with the three-marker tree with reference to the position of most main lineages in Shoreeae (*Hopea*, *Anthoshorea*, *Doona*, *Parashorea,* and *Richetia*) but the phylogenetic relationships of *Rubroshorea* and Balau lineages remained unresolved based on this single marker. Still, *matK* alone was not efficient to correctly place the species within the genus *Hopea*: *H. nervosa* and the specimen *H. myrtifolia* KR4130 showed low differentiation. A similarly dubious position was observed for the sample *S. bracteolata* KR4573, which clustered with low support in the same clade as *Parashorea* (Figure S2).

A lack of resolution was observed in the phylogenetic analysis based on the *rbcL* marker regarding the topology of the main subfamilies (Monotoideae and Dipterocarpoideae) and main sections (Figure S3), while the phylogenetic relationships of these lineages were clarified in the phylogenies based on *matK* (Figure S2) and the concatenated markers (Figure 3 and Figure 4 and Figures S1). Species of genera *Anisoptera* (PP > 0.8), *Vatica* (PP = 1.0) and *H. myrtifolia* (PP = 1.0) clustered with high support at species level (Figure S3). In contrast with the *matK* tree (Figure S2), the genus *Parashorea* fell into the *Rubroshorea* clade with low node support (PP = 0.39) in the *rbcL* tree (Figure S3).

The phylogenetic tree based on the intergenic spacer *trnL-F* was efficient to resolve the relationship only of the species *H. myrtifolia*, *S. bracteolata, S. gibbosa,* and *A. costata,* and presented clear distinction at subfamily level. However, the overall topology of the tree displayed low resolution concerning the position of the main lineages of the Dipterocarpaceae family (Figure S4).

## 4. Discussion

### 4.1. Applicability of DNA Barcoding

We used a dataset of dipterocarp samples from Sumatra to explore the utility of DNA barcoding for species identification in this poorly sampled tropical region and for groups where traditional morphology-based species identification is challenging. Our results show that the applicability of barcoding depends on the chosen markers and the analyzed clades.

The *matK* marker has a high evolutionary rate, which gives a high discriminatory power among angiosperm species [28,47,48]. The phylogenetic trees reconstructed in this study using *matK* had a reasonable resolution to the species-level, giving a broad view of the relationships among Dipterocarpaceae species. Nevertheless, *matK* has been reported to have a lower universality, meaning that it is difficult to amplify specimens from evolutionary distant clades if they are arranged in a high-throughput format with the currently established PCR primers [28,29,48]. The currently established PCR primers for *matK* showed a high rate of recovery within family Dipterocarpaceae, which helped to avoid this problem. In their review about single-locus DNA barcodes, Li et al. [48] indicated that the discrimination rate of *matK* ranges from 49% to 90% across different taxonomic groups.

Contrarily, the *rbcL* marker provides a high universality in terms of steady PCR amplification, high-quality bidirectional sequencing, and reliable nucleotide sequence alignment in most land plants. However, *rbcL* does not have sufficient discriminatory power due the relatively low divergence of this locus in flowering plants observed also in the current study; *rbcL* alone was inefficient to access the main lineages of the Dipterocarpaceae [28,29].

Similar low resolution of the phylogenetic relationships among the main lineages of the Dipterocarpaceae family was observed for the intergenic spacer *trnL-F*. However, the combined use of *rbcL* and *matK* has proven to be a powerful tool in phylogenetic analyses by combining the two strong features of both markers (high levels of polymorphism in *matK* and the universality of *rbcL*) [24,28,29,40] and the applicability of both barcode markers is confirmed by our study for most clades (Figure 3, Figure 4 and Figure S1). The two-marker tree (*matK* + *rbcL*) displayed an equivalent topology to the phylogenetic tree based on the three combined markers (*matK, rbcL,* and *trnL-F*) and it was efficient to access the main lineages with an equal level of resolution (Figure 4). The phylogenetic tree based on the three markers was superior to the two-marker system (*matK* + *rbcL*) only by displaying higher support of the nodes, allowing a better interpretation of the evolutionary history of the group.

### 4.2. Phylogenetic Relationships of Sumatran Dipterocarpaceae

The phylogenetic analysis based on two (*matK* and *rbcL*) and three (*matK*, *rbcL*, and *trnL-F*) DNA barcoding markers confirmed the monophyly of the subfamily Dipterocarpoideae and assembled its main lineages in agreement with previous studies using denser taxon sampling and more markers [23] or phylogenomic methods [3]: Dipterocarpoideae is composed of two major clades. The first clade contains all genera of tribe Dipterocarpeae except the toponymous *Dipterocarpus* – i.e., *Upuna*, *Stemonoporus*, *Anisoptera* (three specimens of one species sampled in our study), *Vatica* (one specimen sampled), *Cotylelobium*, *Vateria*, and *Vateriopsis* (not sampled here) – and corresponds to clade IV of Heckenhauer et al. [23] (Figure 3 and Figure 4). In the second clade, *Dipterocarpus* and *Dryobalanops* are successively sister to the tribe Shoreeae, which contains the bulk of our specimens. The largest genus of Shoreeae, *Shorea*, has been shown to be paraphyletic with respect to the smaller monophyletic genera *Hopea*, *Parashorea*, *Neobalanocarpus*, and *Pentacme* (the latter two not sampled here) based on plastid [1,14,20,23,49] and nuclear markers [17], a combination of both [16], and RADseq [3]. However, when *Shorea* is split into subgenera, monophyletic groups can be retrieved in this tribe [3,14,23,40], and these groups are supported by morphological characters [12,22,36]. The topology of Shoreeae from our study differs somewhat from that of previous studies [3,14,17], mainly in the placement of *Doona* and *Richetia* (Figure 3 and Figure 4), but overall finds the same monophyletic groups: *Hopea*, *Parashorea*, and the *Shorea* subgenera *Anthoshorea* (white meranti), *Doona*, *Richetia* (yellow meranti), *Shorea* (balau), and *Rubroshorea* (red meranti). The paraphyletism of *Shorea* calls for a redefinition of generic boundaries as previously suggested [3,14] in Shoreeae, either including all species of Shoreeae in an expanded *Shorea* s.l. or by raising the mentioned subgenera to generic rank in line with the classifications based on general morphology [12,22,36].

The phylogenetic relationships of the relatively young (evolutionary age ca. 15 Ma [23]) and species rich (68 species [13]) *Rubroshorea* could so far only be reliably resolved by using genomic data, possibly indicating incomplete lineage sorting, which would be consistent with a recent and ongoing diversification of the group and/or adaptive introgression. Most of the species of *Rubroshorea,* for which we had several samples available, were retrieved as polyphyletic, especially in the two-markers tree (*matK* + *rbcL*), but also in the three-markers analysis (Figure 3 and Figure 4). This could indicate mis-identification of species based on morphology. Correct species identification in dipterocarps is hampered by the fact that often, only vegetative material from the specimens is available, as was the case in our study. However, decades of work by dedicated field and herbarium taxonomists have produced vast literature [11,12,13,36] for identification based on vegetative traits of trunk, bark, twigs, stipules, and leaves (Figure 1). Taking into account these traits, species of red meranti (*Rubroshorea*) in Sumatra can be distinguished fairly easily. An exception is the distinction between the two subspecies of *Shorea parvifolia* (Figure 1e–f), which remains challenging as traits are variable depending on the life stage of the trees and because forms that are intermediate between the subspecies can occur. Notably, even morphologically clearly distinct species such as *Shorea ovalis* (Figure 1d) appeared in different clades, so even if we failed in identifying all specimens correctly, a strong mismatch between morphology and the barcoding results for the *Rubroshorea* clade remains. The prevalent low support values in the clade indicate that the markers used in our study do not provide sufficient resolution for species-level identification of red meranti (*Rubroshorea*) taxa. For better results, the inclusion of additional markers or phylogenomic approaches would be desirable. In addition, reference databases are often geographically strongly biased, which may hamper the reliability of phylogenetic trees. With our study, we provide DNA barcodes for 13 species and two subspecies of Dipterocarpaceae from the under-sampled island of Sumatra.

Although DNA barcoding does not always have enough discriminatory power to retrieve species phylogenetically, the present study supports the applicability of the markers *rbcL*, *matK*, and *trnL-F* for placing dipterocarp specimens in highly-supported major clades corresponding to taxonomic groups at the level of genus or subgenus. In addition, in all clades, except *Rubroshorea*, multiple specimens per species were resolved in highly supported monophyletic clades, often clearly distinguished from other congeneric species (Figure 3). These results thus confirm that (with the exception of *Rubroshorea* species) dipterocarp species in Sumatra can reliably be separated using DNA barcoding.

### 4.3. Combined Identification Using Morphology and DNA Barcodes

The use of DNA barcoding allows to recognize taxonomic misidentification of samples and facilitate the identification of phylogenetic species. In this study, DNA barcoding proved to be a useful tool to enhance the accuracy of the taxonomic identification of the taxa belonging to the Dipterocarpaceae family. Especially in the context of plot-based sampling, as applied in the EFForTS project, a large number of collected specimens usually contain juvenile or otherwise vegetative material. Placement of these specimens in the correct major taxonomic groups (families, genera) based on morphology alone is often a great challenge and a time-consuming pursuit. DNA barcoding can greatly facilitate this process. However, it does not replace the traditional taxonomic identification, at least for taxa with high diversification rates or lack of reliable reference data, as shown for the *Rubroshorea* clade. Rather, DNA barcoding and traditional taxonomic approaches complement each other for inventories of diversity. Once a comprehensive and standardized reference database is established for understudied regions such as Sumatra, Indonesia, we advocate that the DNA barcoding method can accelerate taxonomic inventories and species discovery with great precision and be applicable for analysis of phylogenetic diversity.

## 5. Conclusions

The joint use of DNA barcoding markers *rbcL* and *matK* is a reliable tool for identification of land plants from Sumatra, Indonesia and the addition of further markers, such as the *trnL-F* marker, provide better node support of the lineages. Remarkably, the phylogenetic tree based on the DNA barcoding markers employed in this study recovered a topology largely consistent with recent studies based on phylogenomic data [3,19].

In summary, we highlight the applicability of the DNA barcoding as a reliable tool for species inventories and evolutionary studies in tropical areas. Nevertheless, this method cannot be taken as a replacement for taxonomic identification, but should rather be seen as a complementary tool to support the classical taxonomy.

## Figures and Tables

**Table 1:** List of primers used in this study.

Barcode Region	Name of Primer	Primer Sequence (5′ → 3′)	Reference
*matK*	*3F_KIM_f*	CGTACAGTACTTTTGTGTTTACGAG	[28]
	*1R_KIM_r*	ACCCAGTCCATCTGGAAATCTTGGTTC	[28]
	*390_f*	CGATCTATTCATTCAATATTTC	[28,37]
	*990_r*	GGACAATGATCCAATCAAGGC	[14]
*rbcL*	*rbcLa_f*	ATGTCACCACAAACAGAGACTAAAGC	[38,39]
	*rbcLajf634R_r*	GAAACGGTCTCTCCAACGCAT	[40]
*trnL-F*	B49317_f	CGAAATCGGTAGACGCTACG	[30]
	B49873_f	GGTTCAAGTCCCTCTATCCC	[30]
	A50272_r	ATI’TGAACTGGTGACACGAG	[30]

**Table 2:** Sequence information and characteristics of the *rbcL*, *matK* and *trnL*-*F* loci.

Parameter	*matK*	*rbcL*	*trnL-F*
Number of samples used for amplification and sequencing	78	78	78
Number of obtained sequences	66	66	42
Sequencing success rate, %	81.0	83.5	54.0
Variable sites (proportion), %	23.1	21.3	18.0
Parsimony-informative sites (proportion), %	11.1	7.8	-
CG content mean (range), %	32.3	43.7	29.0
Length of the alignment, bp	614	603	987

**Table 3:** Number of taxa genetically resolved for Dipterocarpaceae using individual barcode markers and the combined dataset. Subgenera of *Shorea* s.l. (*Anthoshorea*, *Doona*, *Richetia*, *Shorea* s.s., *Rubroshorea*) are counted as distinct genera.

Barcode Regions	Species	Genera	Subfamily	Family
*matK*	17	12	2	1
*rbcL*	12	11	2	1
*trnL-F*	18	14	2	1
*matK + rbcL*	20	16	2	1
*matK + rbcL + trnL-F*	21	16	2	1

## Supplementary Materials

The following are available online at https://www.mdpi.com/2223-7747/8/11/461/s1, Figure S1: Maximum likelihood tree based on the concatenated sequences of the *matK*, *rbcL*, and *trnL*-*F* markers; Figure S2: Bayesian Inference based on the sequences of the *matK* marker; Figure S3: Bayesian Inference based on the sequences of the *rbcL* marker; Figure S4: Bayesian Inference based on the sequences of the *trnL-F* marker. Table S1: Samples used in the present study collected in Sumatra, Indonesia; and taxonomic identification conducted with and without the use of DNA barcoding dataset.
