# Supplementary material for: Integrating DNA Barcoding and Traditional Taxonomy for the Identification of Dipterocarps in Remnant Lowland Forests of Sumatra
Source: Plants (Basel). 2019 Oct 30;8(11):461. doi: 10.3390/plants8110461 (PMC6918277; doi:10.3390/plants8110461)
Supplement: Supplementary file 1 [file plants-08-00461-s001.zip › Figure S4.pdf]

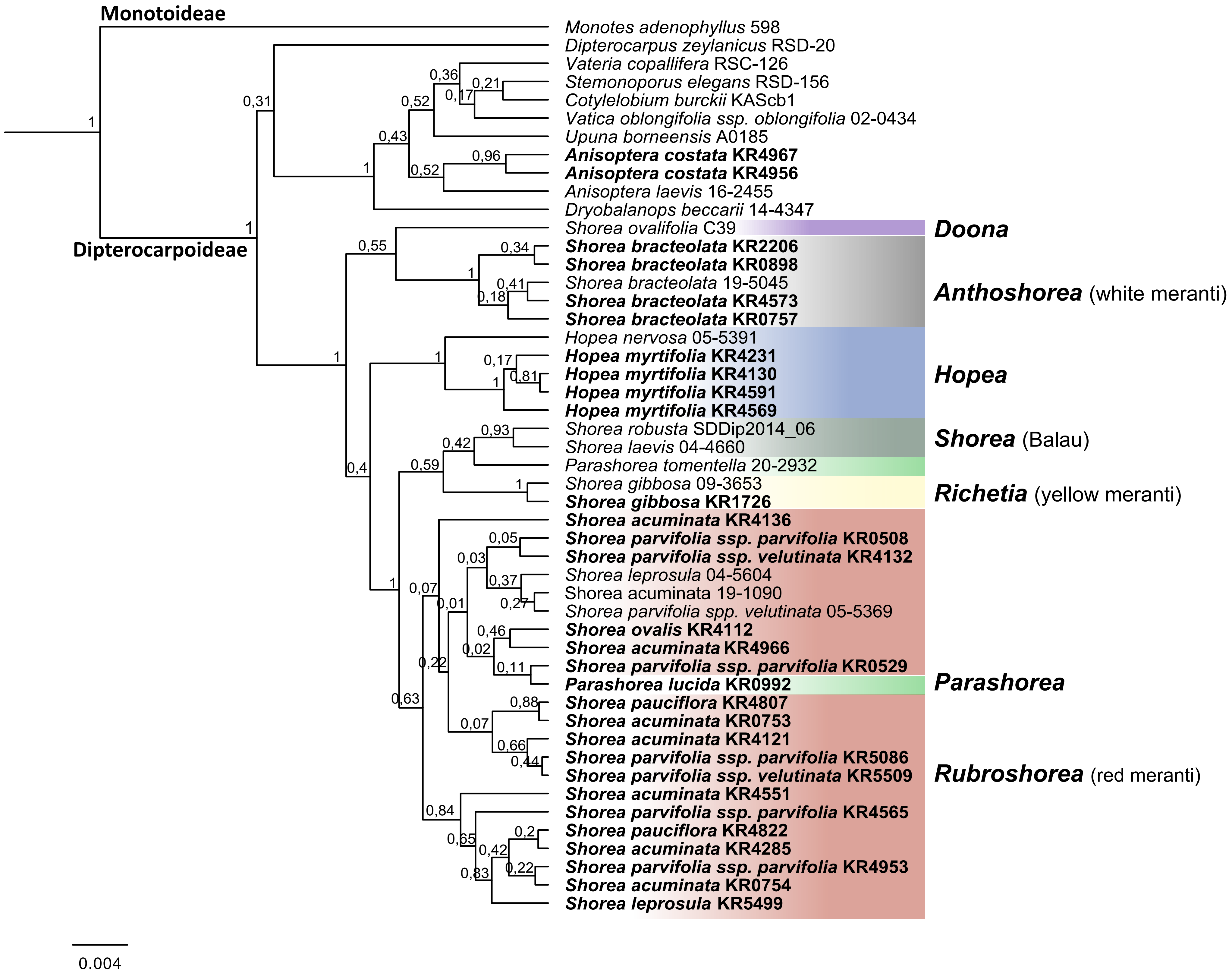
Monotoideae
Dipterocarpoideae
Monotes adenophyllus 598
Dipterocarpus zeylanicus RSD-20
Vateria copallifera RSC-126
Stemonoporus elegans RSD-156
Cotylelobium burckii KAScb1
Vatica oblongifolia ssp. oblongifolia 02-0434
Upuna borneensis A0185
Anisoptera costata KR4967
Anisoptera costata KR4956
Anisoptera laevis 16-2455
Dryobalanops beccarii 14-4347
Shorea ovalifolia C39
Shorea bracteolata KR2206
Shorea bracteolata KR0898
Shorea bracteolata 19-5045
Shorea bracteolata KR4573
Shorea bracteolata KR0757
Hopea nervosa 05-5391
Hopea myrtifolia KR4231
Hopea myrtifolia KR4130
Hopea myrtifolia KR4591
Hopea myrtifolia KR4569
Shorea robusta SDDip2014_06
Shorea laevis 04-4660
Parashorea tomentella 20-2932
Shorea gibbosa 09-3653
Shorea gibbosa KR1726
Shorea acuminata KR4136
Shorea parvifolia ssp. parvifolia KR0508
Shorea parvifolia ssp. velutinata KR4132
Shorea leprosula 04-5604
Shorea acuminata 19-1090
Shorea parvifolia spp. velutinata 05-5369
Shorea ovalis KR4112
Shorea acuminata KR4966
Shorea parvifolia ssp. parvifolia KR0529
Parashorea lucida KR0992
Shorea pauciflora KR4807
Shorea acuminata KR0753
Shorea acuminata KR4121
Shorea parvifolia ssp. parvifolia KR5086
Shorea parvifolia ssp. velutinata KR5509
Shorea acuminata KR4551
Shorea parvifolia ssp. parvifolia KR4565
Shorea pauciflora KR4822
Shorea acuminata KR4285
Shorea parvifolia ssp. parvifolia KR4953
Shorea acuminata KR0754
Shorea leprosula KR5499
Doona
Anthoshorea (white meranti)
Hopea
Shorea (Balau)
Richetia (yellow meranti)
Parashorea
Rubroshorea (red meranti)
0.004
