# Supplementary figures and images for: Integrating DNA Barcoding and Traditional Taxonomy for the Identification of Dipterocarps in Remnant Lowland Forests of Sumatra
Source: Plants (Basel). 2019 Oct 30;8(11):461. doi: 10.3390/plants8110461 (PMC6918277; doi:10.3390/plants8110461)

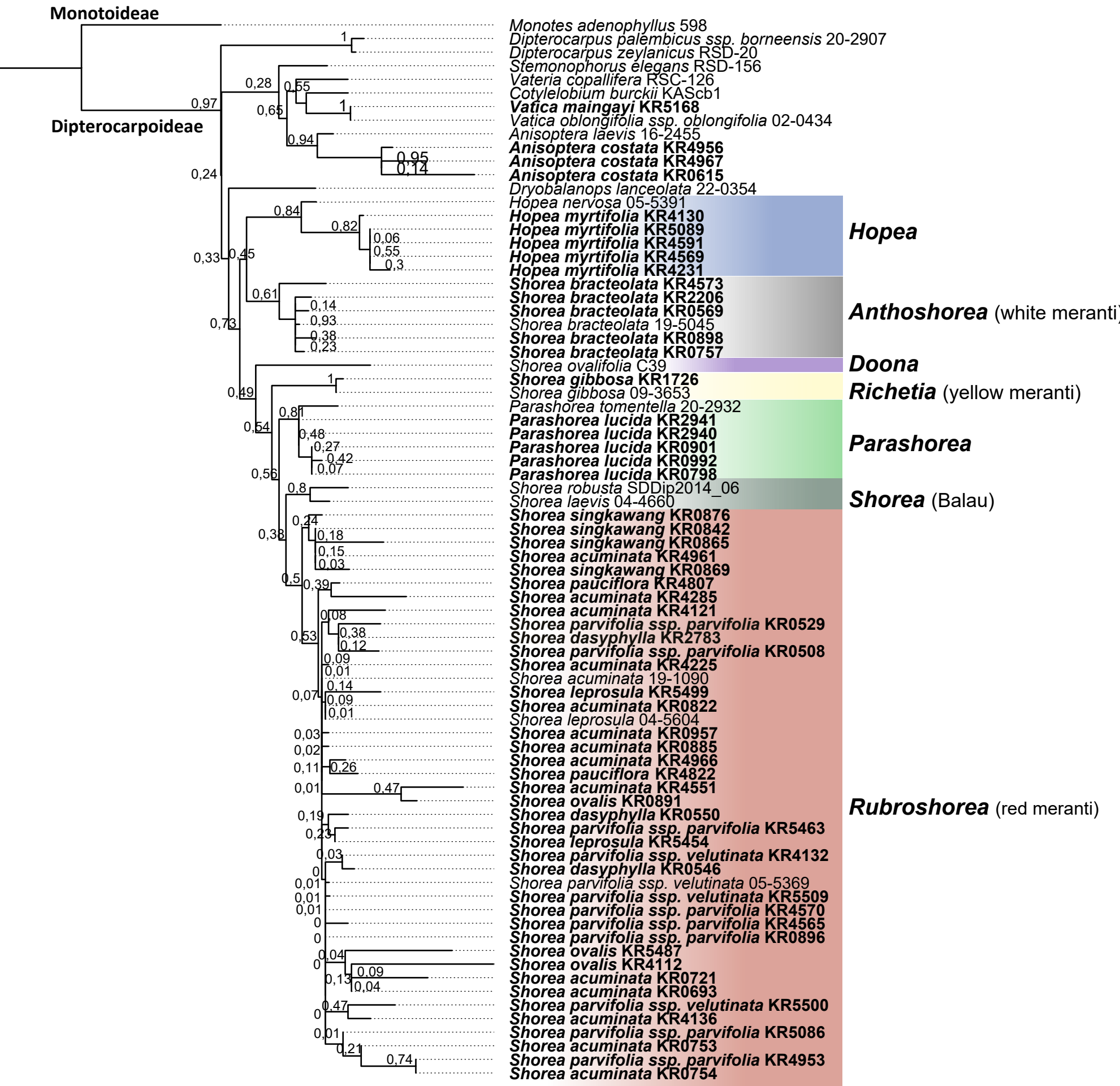

Supplement: Supplementary file 1 [file plants-08-00461-s001.zip › Figure S1.pdf]

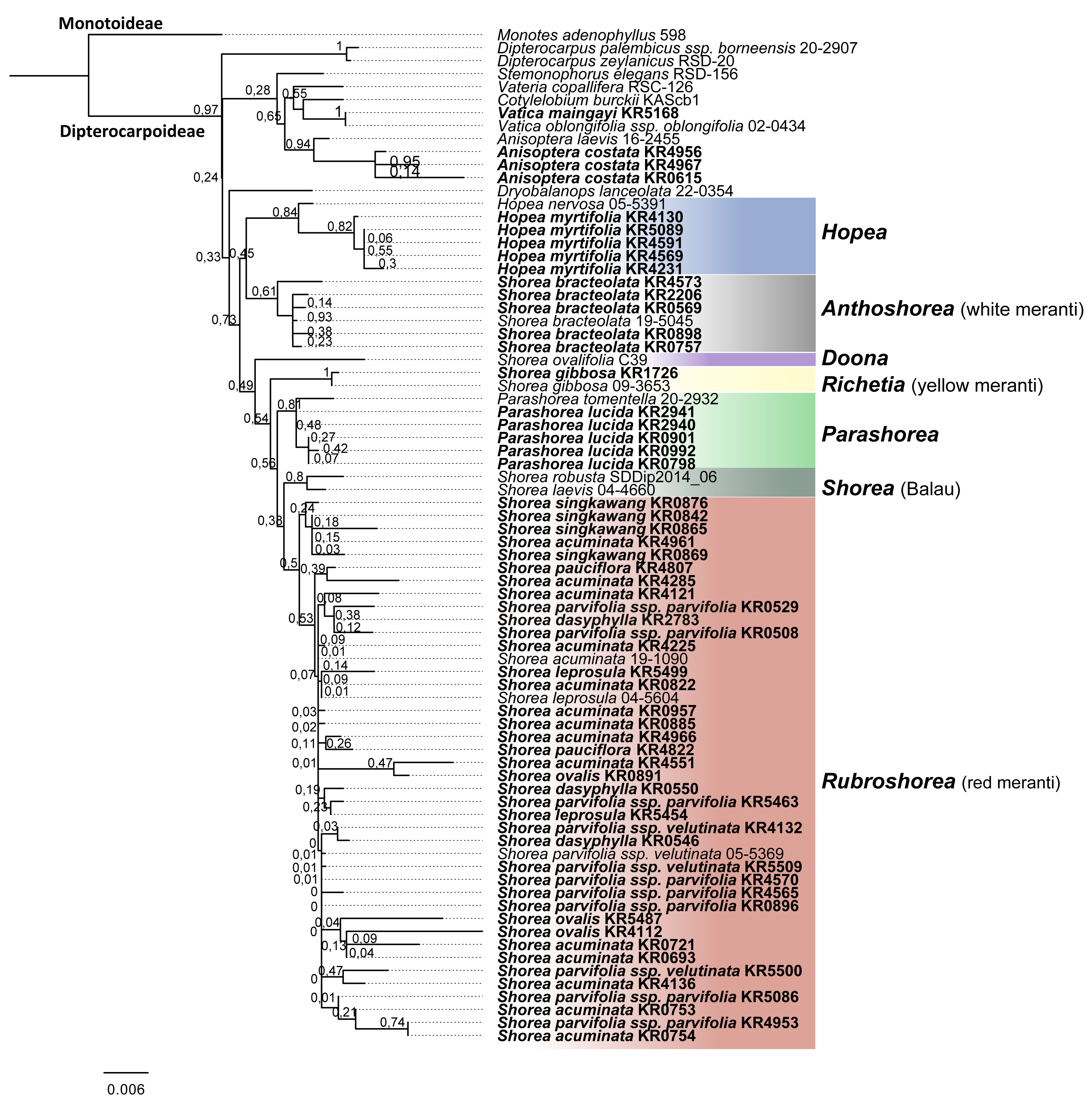

Supplement: Supplementary file 1 [file plants-08-00461-s001.zip › Figure S1.tif]

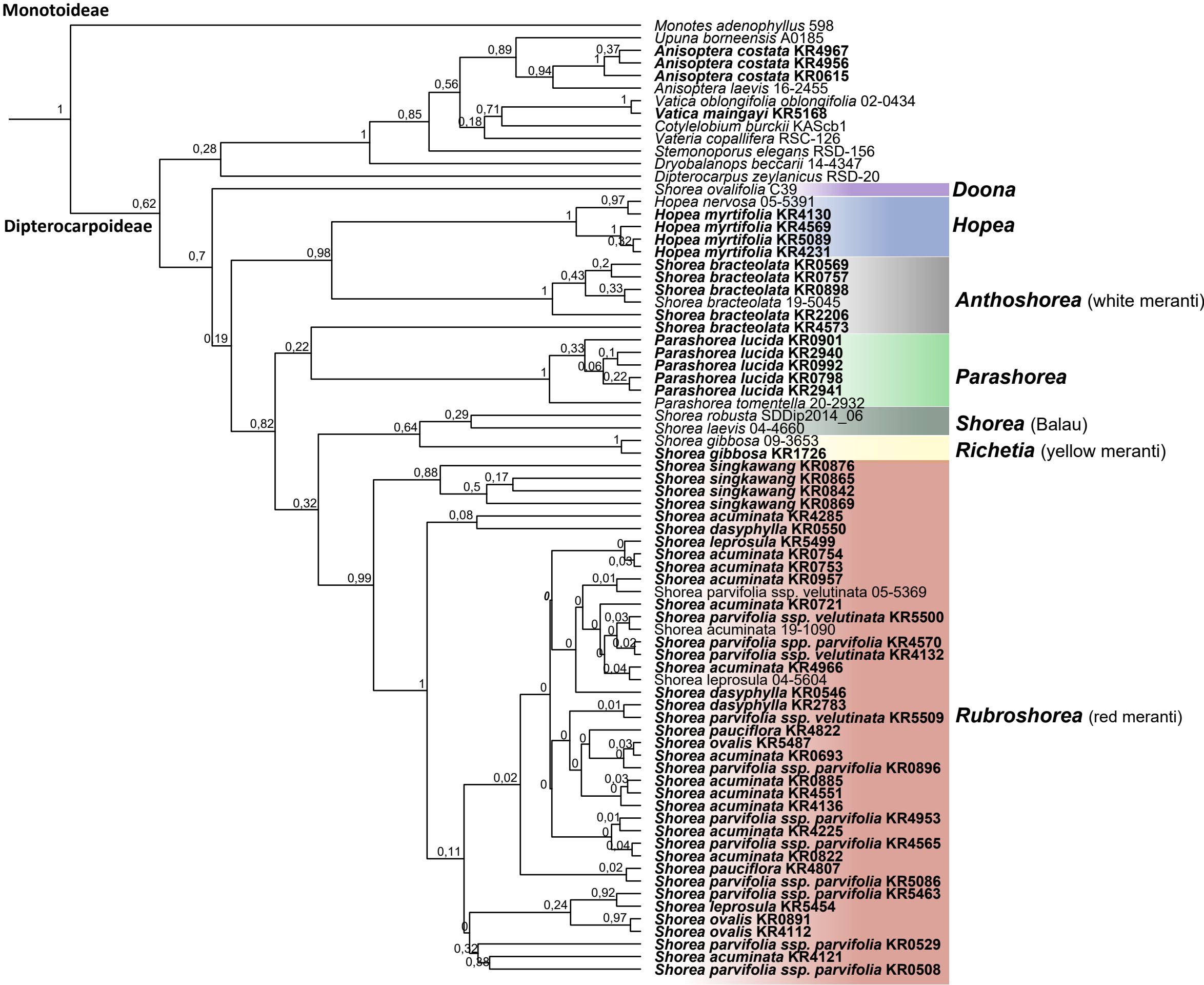

Supplement: Supplementary file 1 [file plants-08-00461-s001.zip › Figure S2.pdf]

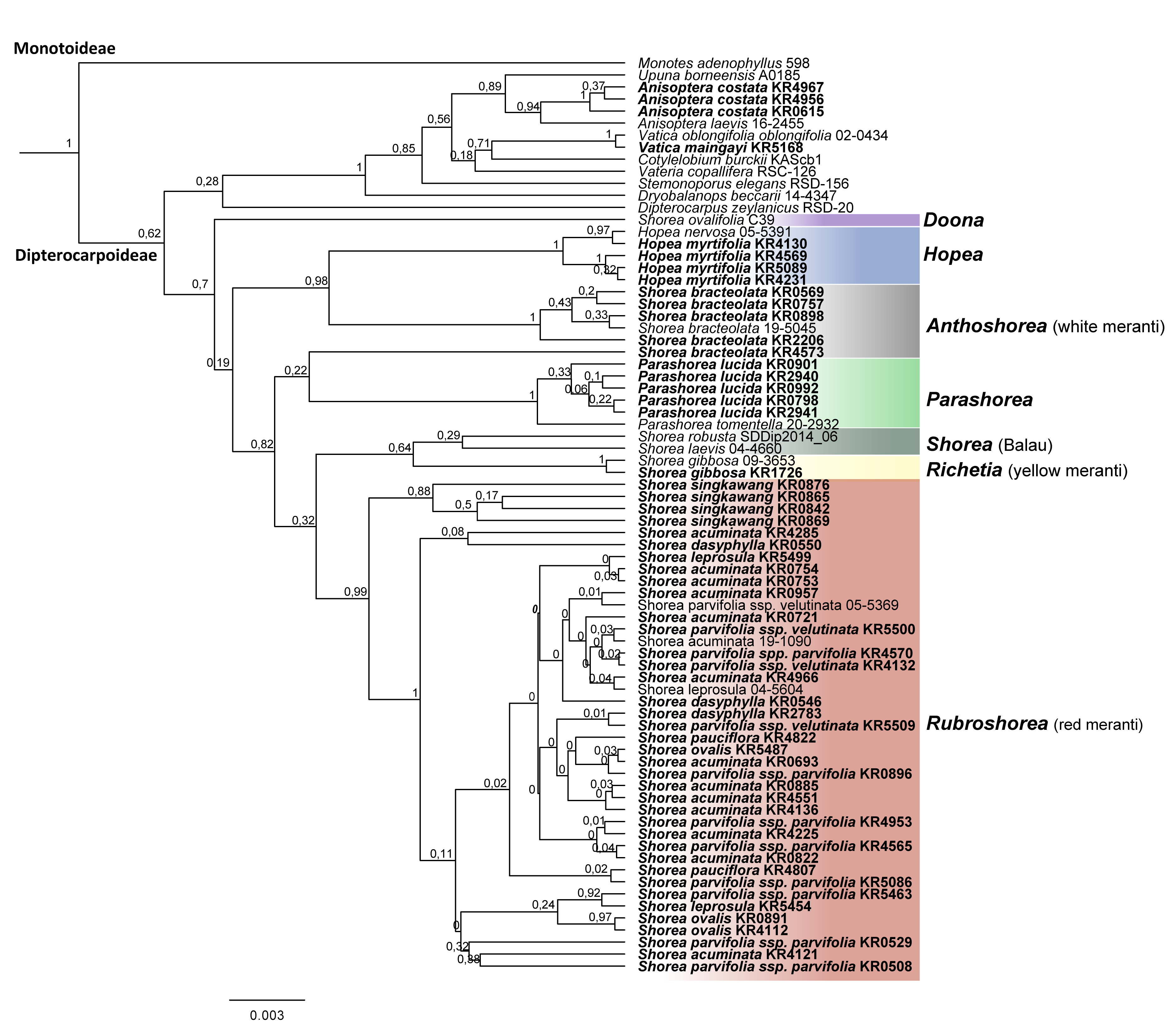

Supplement: Supplementary file 1 [file plants-08-00461-s001.zip › Figure S2.tif]

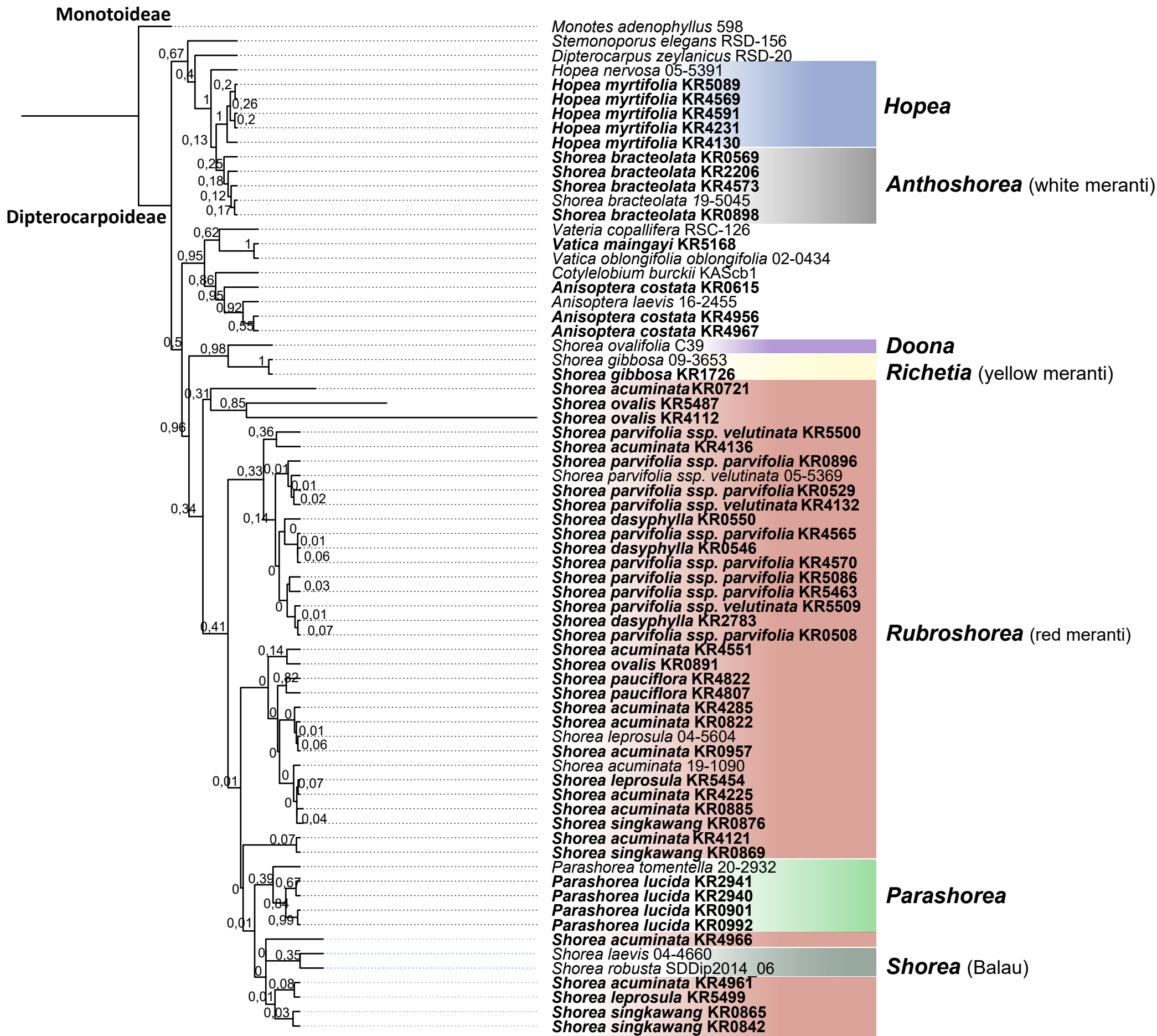

Supplement: Supplementary file 1 [file plants-08-00461-s001.zip › Figure S3.pdf]

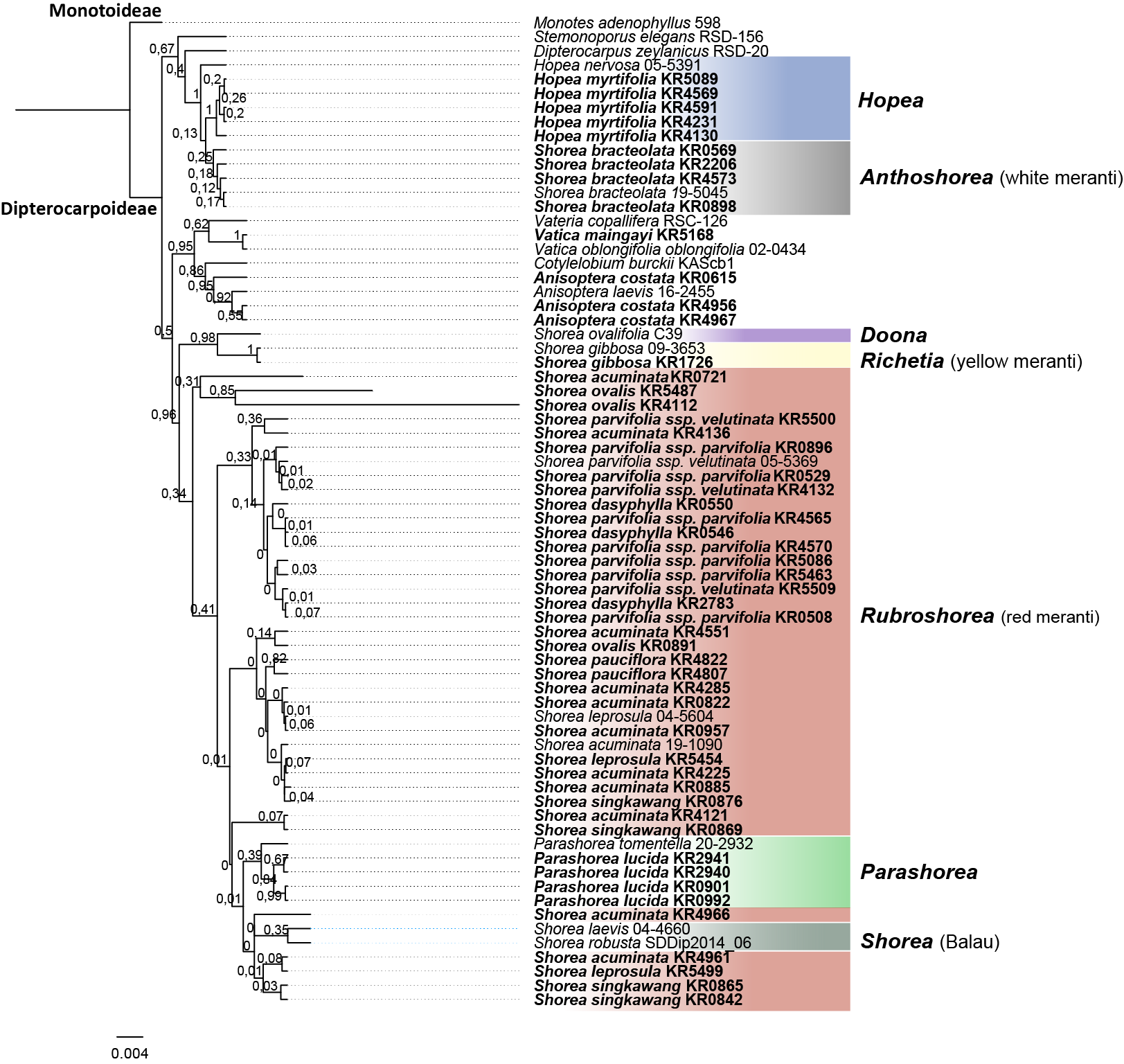

Supplement: Supplementary file 1 [file plants-08-00461-s001.zip › Figure S3.tif]

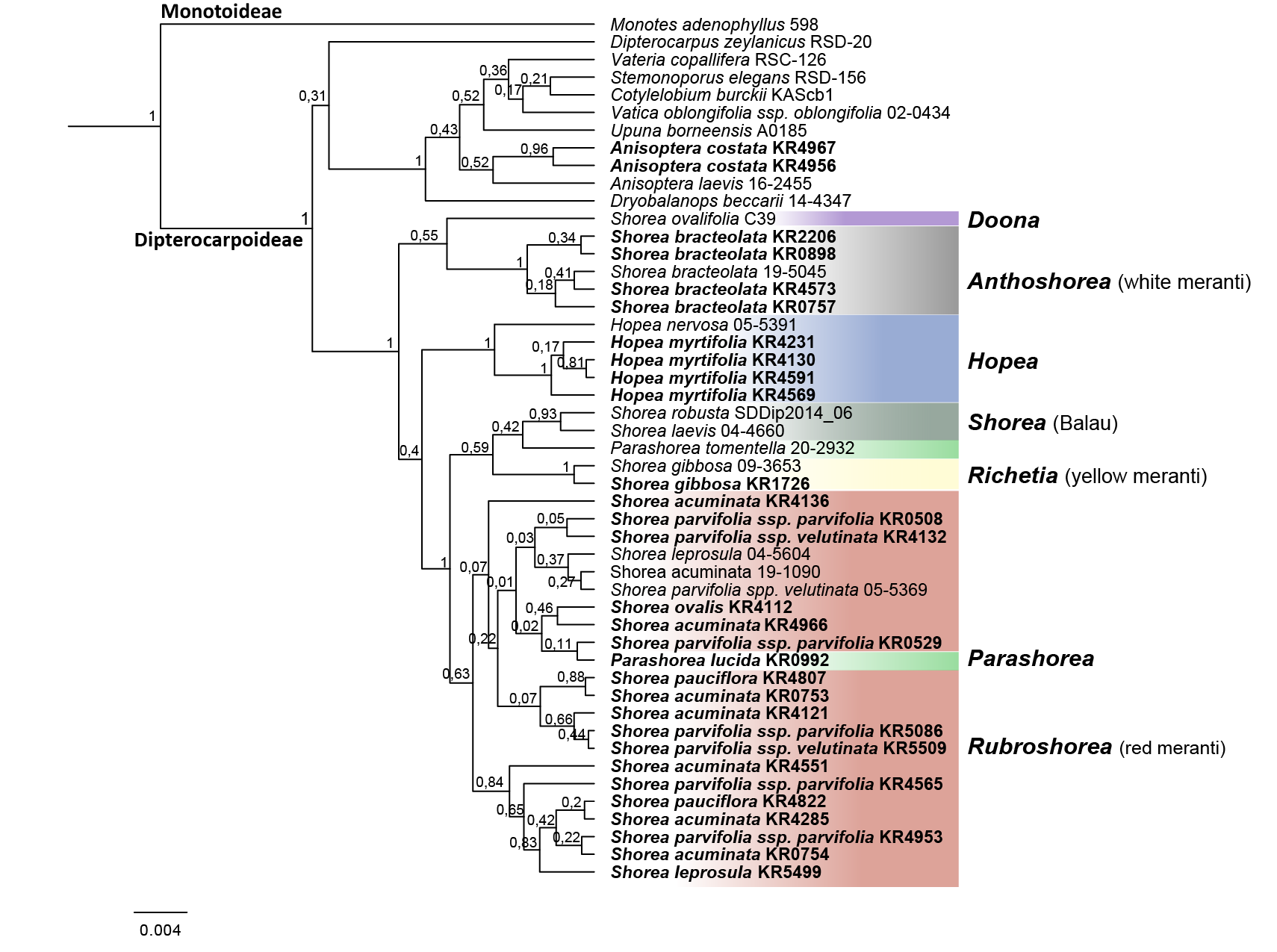

Supplement: Supplementary file 1 [file plants-08-00461-s001.zip › Figure S4.tif]
